# Retraction Note to: Integrated physiological and proteomic analysis of embryo and endosperm reveals central salt stress response proteins during seed germination of winter wheat cultivar Zhengmai 366

**DOI:** 10.1186/s12870-019-2019-0

**Published:** 2019-09-17

**Authors:** Dongmiao Liu, Caixia Han, Xiong Deng, Yue Liu, Nannan Liu, Yueming Yan

**Affiliations:** 1 Laboratory of Molecular Genetics and Proteomics, College of Life Science, Capital Normal University, Beijing, 100048 China; 2 Hubei Collaborative Innovation Center for Grain Industry (HCICGI), Yangtze University, Jingzhou, 434025 China

**Retraction note: BMC Plant Biology(2019) 19:29**

**https://doi.org/10.1186/s12870-019-1643-z**

The editor has retracted this article [1] because parts of Figs. 1 and 4 were duplicated from a previously published paper by the same authors [2] without appropriate disclosure. None of the authors have responded to any correspondence from the editor about publication of this retraction notice.
